# Supplementary material for: Resolving the full spectrum of human genome variation using Linked-Reads
Source: Genome Res. 2019 Apr;29(4):635–45. doi: 10.1101/gr.234443.118 (PMC6442396; doi:10.1101/gr.234443.118)
Supplement: Supplemental Material [file supp_gr.234443.118_Supplemental_longranger-2.2.zip › longranger-2.2.tar.bz2!/lr2.2-public/lib/stan/cmdstan/stan/doc/api/html/structstan_1_1gm_1_1contains__var.html]

Stan: stan::gm::contains\_var Struct Reference


|  |  |
| --- | --- |
|  | Stan  2.5.0  probability, sampling & optimization |


- Main Page
- Related Pages
- Namespaces
- Classes
- Files

- Class List
- Class Index
- Class Hierarchy
- Class Members

All Classes Namespaces Files Functions Variables Typedefs Enumerations Enumerator Friends Macros Pages

- stan
- gm
- contains\_var

Public Member Functions |
Public Attributes |
List of all members

stan::gm::contains\_var Struct Reference

`#include <ast.hpp>`

Inheritance diagram for stan::gm::contains\_var:

|  |  |
| --- | --- |
| Public Member Functions | |
|  | contains\_var (const variable\_map &var\_map) |
|  | |
| bool | operator() (const nil &e) const |
|  | |
| bool | operator() (const int\_literal &e) const |
|  | |
| bool | operator() (const double\_literal &e) const |
|  | |
| bool | operator() (const array\_literal &e) const |
|  | |
| bool | operator() (const variable &e) const |
|  | |
| bool | operator() (const integrate\_ode &e) const |
|  | |
| bool | operator() (const fun &e) const |
|  | |
| bool | operator() (const index\_op &e) const |
|  | |
| bool | operator() (const binary\_op &e) const |
|  | |
| bool | operator() (const unary\_op &e) const |
|  | |

|  |  |
| --- | --- |
| Public Attributes | |
| const variable\_map & | var\_map\_ |
|  | |

## Detailed Description

Definition at line 850 of file ast.hpp.

## Constructor & Destructor Documentation

|  |  |  |  |  |  |
| --- | --- | --- | --- | --- | --- |
| stan::gm::contains\_var::contains\_var | ( | const variable\_map & | *var\_map* | ) |  |

Definition at line 597 of file ast\_def.cpp.

## Member Function Documentation

|  |  |  |  |  |  |
| --- | --- | --- | --- | --- | --- |
| bool stan::gm::contains\_var::operator() | ( | const nil & | *e* | ) | const |

Definition at line 600 of file ast\_def.cpp.

|  |  |  |  |  |  |
| --- | --- | --- | --- | --- | --- |
| bool stan::gm::contains\_var::operator() | ( | const int\_literal & | *e* | ) | const |

Definition at line 603 of file ast\_def.cpp.

|  |  |  |  |  |  |
| --- | --- | --- | --- | --- | --- |
| bool stan::gm::contains\_var::operator() | ( | const double\_literal & | *e* | ) | const |

Definition at line 606 of file ast\_def.cpp.

|  |  |  |  |  |  |
| --- | --- | --- | --- | --- | --- |
| bool stan::gm::contains\_var::operator() | ( | const array\_literal & | *e* | ) | const |

Definition at line 609 of file ast\_def.cpp.

|  |  |  |  |  |  |
| --- | --- | --- | --- | --- | --- |
| bool stan::gm::contains\_var::operator() | ( | const variable & | *e* | ) | const |

Definition at line 615 of file ast\_def.cpp.

|  |  |  |  |  |  |
| --- | --- | --- | --- | --- | --- |
| bool stan::gm::contains\_var::operator() | ( | const integrate\_ode & | *e* | ) | const |

Definition at line 627 of file ast\_def.cpp.

|  |  |  |  |  |  |
| --- | --- | --- | --- | --- | --- |
| bool stan::gm::contains\_var::operator() | ( | const fun & | *e* | ) | const |

Definition at line 621 of file ast\_def.cpp.

|  |  |  |  |  |  |
| --- | --- | --- | --- | --- | --- |
| bool stan::gm::contains\_var::operator() | ( | const index\_op & | *e* | ) | const |

Definition at line 633 of file ast\_def.cpp.

|  |  |  |  |  |  |
| --- | --- | --- | --- | --- | --- |
| bool stan::gm::contains\_var::operator() | ( | const binary\_op & | *e* | ) | const |

Definition at line 636 of file ast\_def.cpp.

|  |  |  |  |  |  |
| --- | --- | --- | --- | --- | --- |
| bool stan::gm::contains\_var::operator() | ( | const unary\_op & | *e* | ) | const |

Definition at line 640 of file ast\_def.cpp.

## Member Data Documentation

const variable\_map& stan::gm::contains\_var::var\_map\_

Definition at line 851 of file ast.hpp.

---

The documentation for this struct was generated from the following files:

- src/stan/gm/ast.hpp
- src/stan/gm/ast\_def.cpp


---

[ Stan Home Page ]

*© 2011–2014,
Stan Development Team.*
